# The Avian Transcriptome Response to Malaria Infection

**DOI:** 10.1093/molbev/msv016

**Published:** 2015-01-29

**Authors:** Elin Videvall, Charlie K. Cornwallis, Vaidas Palinauskas, Gediminas Valkiūnas, Olof Hellgren

**Affiliations:** ^1^Department of Biology, Lund University, Lund, Sweden; ^2^Institute of Ecology, Nature Research Centre, Vilnius, Lithuania

**Keywords:** host response, immune defense, Plasmodium, transcriptome, experimental infection, RNA-seq

## Abstract

Malaria parasites are highly virulent pathogens which infect a wide range of vertebrates. Despite their importance, the way different hosts control and suppress malaria infections remains poorly understood. With recent developments in next-generation sequencing techniques, however, it is now possible to quantify the response of the entire transcriptome to infections. We experimentally infected Eurasian siskins (*Carduelis spinus*) with avian malaria parasites (*Plasmodium ashfordi*), and used high-throughput RNA-sequencing to measure the avian transcriptome in blood collected before infection (day 0), during peak parasitemia (day 21 postinfection), and when parasitemia was decreasing (day 31). We found considerable differences in the transcriptomes of infected and uninfected individuals, with a large number of genes differentially expressed during both peak and decreasing parasitemia stages. These genes were overrepresented among functions involved in the immune system, stress response, cell death regulation, metabolism, and telomerase activity. Comparative analyses of the differentially expressed genes in our study to those found in other hosts of malaria (human and mouse) revealed a set of genes that are potentially involved in highly conserved evolutionary responses to malaria infection. By using RNA-sequencing we gained a more complete view of the host response, and were able to pinpoint not only well-documented host genes but also unannotated genes with clear significance during infection, such as microRNAs. This study shows how the avian blood transcriptome shifts in response to malaria infection, and we believe that it will facilitate further research into the diversity of molecular mechanisms that hosts utilize to fight malaria infections.

## Introduction

Malaria parasites (*Plasmodium* spp.) are intracellular apicomplexan protists that are most commonly known because they cause the disease malaria in an estimated 200 million people every year, resulting in over 600,000 deaths (WHO 2013). These parasites are transmitted through dipteran vectors to an extremely diverse range of vertebrate hosts including primates, bats, rodents, reptiles, and birds (Garnham 1966; Levine 1988). Most of the research on host responses to malaria have been performed on primates and mice, with the effects of *Plasmodium* on other vertebrate hosts and the mechanisms they use to deal with infections remaining largely unknown.

To understand why some hosts are greatly affected by malaria while others have evolved different mechanisms to resist or tolerate infections (Råberg et al. 2009), it is imperative to characterize the molecular responses to infection across different taxa. To date, there has been little integration between different host systems and between wild and laboratory (model) organisms. Most of the research on molecular host responses to malaria have concentrated on humans and laboratory mice. In contrast, studies on wild populations, which are mainly on birds, have focused largely on ecological factors associated with distribution patterns, and hence the diversity of molecular mechanisms underlying infections is still mostly unknown. However, investigating the molecular responses and immune mechanisms of birds has several potential advantages. The way natural selection shapes the evolution of host responses can be studied without interference by vaccinations, medicines, mosquito control, and other anthropogenic preventive measures. It is also possible to perform controlled malaria experiments in wild birds in order to follow host responses over the course of infections. Inbred laboratory mice strains are well suited for infection experiments, although they have been artificially selected and cannot accurately represent natural host responses in other vertebrate taxa, including humans (White et al. 2010). Studying the molecular responses of wild birds to malaria infections therefore presents a unique opportunity to better understand the ecological and evolutionary basis of molecular host responses. Considering that many major malaria discoveries have historically been made in species other than humans (Cox 2010), several of them in birds, studies encompassing a broader host diversity are likely to provide important advances in malaria research.

Birds represent a major host group to *Plasmodium*. Around 50 morphological avian malaria parasite species have been described (Valkiūnas 2005), but over 500 evolutionary independent lineages have been discovered using sequence divergence in the mitochondrial cytochrome *b* gene (Bensch et al. 2004, 2009). Several *Plasmodium* species infect numerous bird species and have a near global distribution, whereas others are more host specific with limited transmission (Ricklefs and Fallon 2002; Bensch et al. 2009; Hellgren et al. 2009). Their virulence often varies substantially both within and between host species, and interestingly, some birds seem to be resistant to malaria parasites (Palinauskas et al. 2008, 2011). The disease symptoms in birds range from asymptomatic life-long chronic levels without apparent fitness effects on the host (Bensch et al. 2007), to severe anemia, cerebral paralysis, and even death (Swann and Kreier 1973; Atkinson et al. 1995, 2000; Palinauskas et al. 2008; Cellier-Holzem et al. 2010). They have also been shown to inflict drastic consequences on wild populations; for example, *Plasmodium relictum* has caused major declines and the extinction of several endemic bird species on the islands of Hawaii (van Riper et al. 1986; Atkinson and LaPointe 2009).

Studying the molecular mechanisms of host immune responses to malaria infection in general is extremely challenging. The immune defense of vertebrates is highly diverse and complex, with numerous cells, proteins, genes, and pathways, interacting and activating various components of the immunity. Previous methods used to characterize host molecular responses to malaria parasites have focused on microarrays and/or various genetic techniques targeting a set number of candidate genes. However, these tools rely heavily upon already existing knowledge of the gene sequences and are limited in their ability to comprehensively monitor and accurately quantify host transcriptional response (Wang et al. 2009; Oshlack et al. 2010; Westermann et al. 2012). This makes it difficult to gain a holistic picture of host genes activated during different stages of infection, particular in nonmodel organisms, and it prevents potential discoveries of novel genes and pathways with important roles during infection. With the advent of next-generation sequencing methods, we now have the unprecedented opportunity to accurately evaluate the overall molecular response to infection in a wide range of taxa. One of these new sequencing techniques is RNA-sequencing (RNA-seq), where total mRNA is sequenced and analyzed with bioinformatic approaches, resulting in a complete representation of the transcriptome.

**Fig. 1.**
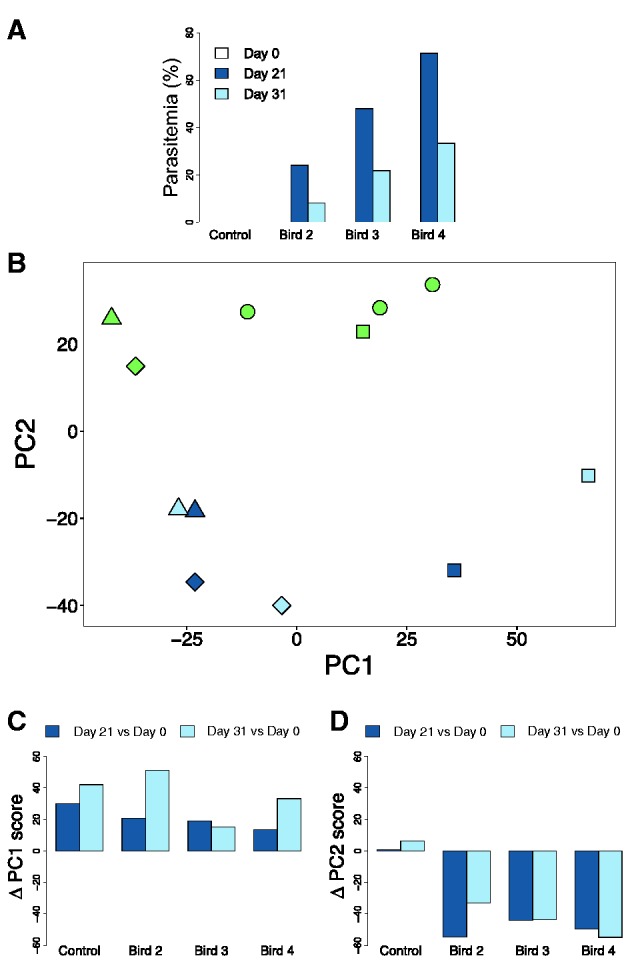
The avian transcriptome shifts in response to malaria infection. (*A*) Parasitemia levels (% infected red blood cells) in one control bird and three inoculated birds at day 0 (before infection), day 21 (during peak parasitemia), and day 31 (during decreasing parasitemia). (*B*) Principal component (PC) analysis plot showing the relative location of 12 complete transcriptome samples explained by the first two PC dimensions, where distances between samples correspond to a measure of expression similarity for all genes. Uninfected transcriptomes (green) separate from infected transcriptomes during peak parasitemia (dark blue) and decreasing parasitemia (light blue) on the PC2 axis. Symbols represent the different bird individuals; circle = bird 1 (control bird), square = bird 2 (low parasitemia), triangle = bird 3 (intermediate parasitemia), and diamond = bird 4 (high parasitemia). (*C*) Change in PC1 scores over time for the different individuals. All birds, including the control, move in a positive direction in the PC1 dimension over the course of the experiment. (*D*) Change in PC2 scores over time for the different individuals. All infected birds, but not the control bird, move in a negative direction in the PC2 dimension over the course of the experiment.

In this study, we examined the response of the avian transcriptome to experimental malaria infections in vivo using RNA-seq. We caught wild malaria-naïve Eurasian siskins (*Carduelis spinus*) and housed them in aviaries throughout the course of the experiment. Three birds were injected once with blood containing *Plasmodium ashfordi* parasites, and a control bird received only blood. We collected blood from the birds at three time points: Before infection (day 0), during the acute stage of infection (peak parasitemia; day 21), and when parasitemia was decreasing (day 31) (fig. 1*A*). RNA-seq data on whole-blood transcriptomes across the three time points were used in the following way. First, we characterized overall transcriptome changes in relation to malaria infection and tested for differentially expressed genes between infected and uninfected birds. Second, we examined whether there were differences in gene expression between the peak and decreasing parasitemia stages. Third, the functions of the differentially expressed genes were determined by investigating annotation status and performing gene set enrichment analyses (GSEA), where we tested for overrepresentation among gene ontology (GO) terms. Finally, we compared the differentially expressed genes in this study with genes found to be associated with malaria infection in humans and mice to identify potentially conserved gene responses across evolutionary divergent taxa.

## Results and Discussion

### The Avian Transcriptome Shifts in Response to Infection

We examined whether there were overall transcriptome differences between infected and uninfected samples using a multidimensional scaling approach in the form of a principal component analysis (PCA). All individuals, including the control bird, moved in a positive direction along the PC1 axis with approximately similar magnitudes over the course of the experiment (fig. 1*B* and *C*). This, together with additional evidence discussed in Materials and Methods, indicated that PC1 captured transcriptome-wide expression variation caused by the effects of experimental procedure and time, but not by malaria infection. PC2, in contrast, represented expression variation between infected and uninfected birds (fig. 1*B* and *D*), that is, the response to malaria. The transcriptomes from all infected samples were well separated from the uninfected ones on the PC2 axis (fig. 1*B*), implying similar transcriptome responses to malaria in all infected birds and during both parasitemia stages. Consequently, the PCA indicated that there had been a considerable shift in the blood transcriptomes of infected birds due to malaria, and that the effects of experimentation were clearly distinguishable from the effects of infection.

**Fig. 2.**
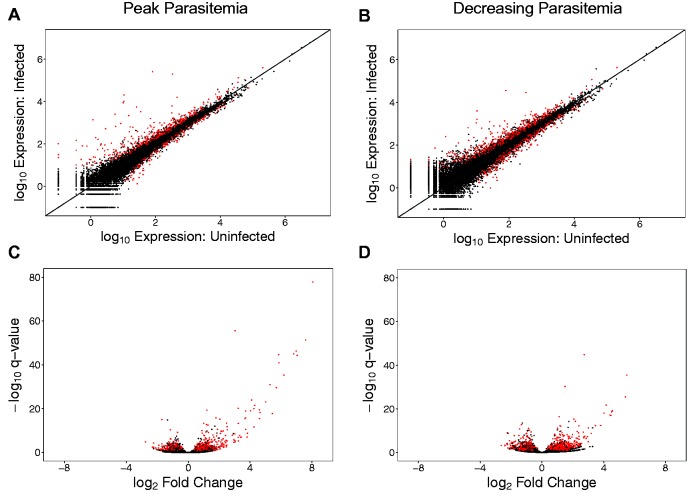
Differentially expressed genes in birds with malaria. (*A*, *B*) Scatter plots with mean normalized gene expression levels (+0.1) in inoculated birds on a log_10_ scale comparing (*A*) peak parasitemia against day 0 (uninfected), and (*B*) decreasing parasitemia against day 0 (uninfected). (C, D) Volcano plots displaying log_2_ fold change in gene expression of (*C*) peak parasitemia/uninfected and (*D*) decreasing parasitemia/uninfected against −log_10_ *q* value. All genes in the transcriptome are shown as points (*n* = 18,618) in all panels, with nonsignificant genes in black and significantly differentially expressed genes in red. A positive log_2_ fold change means that genes are upregulated in infected birds, a negative log_2_ fold change shows downregulated genes in infected birds, and a higher −log_10_ *q* value denotes increased significance.

Next, we performed differential gene expression analyses to identify genes that significantly contributed to the transcriptome-wide differences between infected and uninfected birds. First, we examined changes in gene expression during peak parasitemia by doing pairwise comparisons of expression levels in inoculated birds at day 0 (uninfected) to the same birds at day 21 postinfection (peak parasitemia). In order to separate the effects of malaria infection from the effects of experimentation, we used information gained from the PCA (fig. 1). From the set of genes found to be differentially expressed, we only selected those with PC2 loading scores greater than PC1 loading scores (hereafter referred to as PC2 filtering) (see details in Materials and Methods). After this filtering step, we found a total of 795 significantly differentially expressed genes during peak parasitemia that loaded more strongly onto PC2 than PC1, representing the response to malaria (fig. 2*A* and supplementary table S1, Supplementary Material online). Of these 795 significant genes, almost two-thirds (*n* = 516; 64.9%) were upregulated during infection (fig. 2*A* and *C*), with some genes exhibiting very high log_2_ fold changes (up to 8.0) (fig. 2*C*).

Second, we looked for genes that were differentially expressed during the decreasing parasitemia stage, by pairwise comparisons between inoculated birds before infection (day 0) with the same birds at day 31 after infection. Employing the same PC2 filtering method as in the previous analysis, we found a total of 1,106 genes that responded significantly to malaria (fig. 2*B* and supplementary table S2, Supplementary Material online). Similarly to the results of peak parasitemia, more of these genes were found to be upregulated during infection (*n* = 654; 59.1%) (fig. 2*B* and *D*). The most highly expressed genes during all time points were (in order of expression levels) hemoglobin alpha 1 (*HBAA*), beta-globin, hemoglobin alpha 2 (*HBAD*), and ferritin (*FTH1*); all red blood cell house-keeping genes.

### Transcriptome Similarities between the Two Stages of Parasitemia

In order to investigate similarities and differences in the transcriptional response of the two parasitemia stages, we tested them against each other in a differential gene expression analysis, with subsequent PC2-filtering to rule out the effects of time and experimentation as explained previously. We found that only 23 genes were significantly differentially expressed between days 21 and 31 (supplementary fig. S1 and table S3, Supplementary Material online). This result suggests that gene expression levels were very similar during the two infected time points, despite the much lower parasitemia in the birds 10 days later during the decreasing parasitemia stage (fig. 1*A*). Interestingly, apart from one gene, all of the significant genes had higher expression levels during peak parasitemia (*n* = 22; 95.7%) (supplementary table S3, Supplementary Material online).

**Fig. 3.**
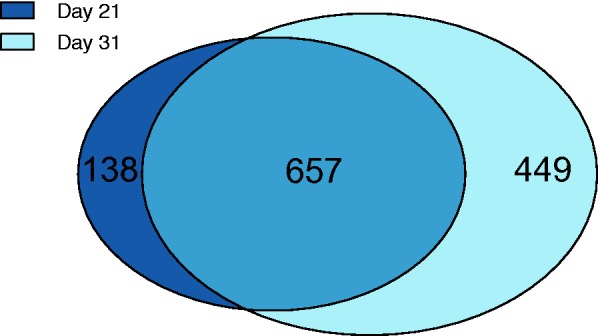
The majority of differentially expressed genes are significant during both parasitemia stages. Venn diagram indicating overlap of significantly differentially expressed genes found during peak parasitemia (dark blue) and decreasing parasitemia (light blue).

To further evaluate similarities between the two parasitemia stages, we examined the overlap of significantly differentially expressed genes found during each time point of infection. The majority of genes that were significant during peak parasitemia were also found to be significant during the decreasing parasitemia stage (82.6%; *n* = 657) (fig. 3). The similarities between the two infected time points were not only qualitative with respect to what genes were found significant, but also quantitative in the sense that the expression levels of all the significant genes were highly correlated (Pearson’s correlation test: *R*_p_ = 0.84; *n* = 1,244; *P* < 2.2e-16) (supplementary fig. S2, Supplementary Material online). Additionally, of the top ten significant genes during peak parasitemia, nine genes remained in the top ten significant genes during decreasing parasitemia as well (table 1).

**Table 1.** Top Ten Most Significant Genes during Peak and Decreasing Parasitemia Stages.

		*q* Values		Expression Levels		
Gene ID	Gene Description	Peak Para.	Decr. Para.	Day 0	Peak Para.	Decr. Para.
ENSTGUG00000017658	miRNA gene	(1) 8.6e-79	(2) 1.9e-36	10.5	10,262.8	1,599.6
ENSTGUG00000009618	Uncharacterized gene (H0ZH49_TAEGU)	(2) 1.5e-56	(1) 8.6e-46	72.4	640.9	513.8
ENSTGUG00000018618	miRNA gene	(3) 2.5e-52	(4) 1.9e-26	10.5	20,433.7	3,871.6
ENSTGUG00000018567	miRNA gene	(4) 2.8e-47	(7) 1.5e-19	332.4	195,011.7	28,144.1
ENSTGUG00000018584	miRNA gene	(5) 5.0e-46	(6) 4.5e-20	1.1	1,366.3	246.0
ENSTGUG00000013390	Calsequestrin 2 (cardiac muscle)	(6) 1.2e-45	(5) 1.1e-22	14.7	2,169.4	606.3
ENSTGUG00000018598	miRNA gene	(7) 2.6e-45	(10) 6.1e-18	10.0	8,694.8	1,092.6
ENSTGUG00000005129	Uncharacterized gene (H0Z3X9_TAEGU)	(8) 6.7e-42	(8) 5.8e-19	2.8	518.4	123.2
ENSTGUG00000008711	Uncharacterized gene (H0ZEF6_TAEGU)	(9) 2.8e-36	(9) 4.1e-18	5.6	2,534.3	642.8
ENSTGUG00000009844	Uncharacterized gene (H0ZHT6_TAEGU)	(10) 6.9e-32	(16) 2.9e-13	5.8	670.0	164.1
ENSTGUG00000012391	Microsomal glutathione S-transferase 1	(18) 2.8e-20	(3) 3.2e-31	1,566.4	3,641.2	4,573.6

Note.—Peak Para., peak parasitemia stage; Decr. Para., decreasing parasitemia stage; Day 0, before infection. Numbers in parentheses indicate order of significance, where 1 is the most significant gene during respective parasitemia stage. For more information about these genes, see supplementary tables S1 and S2, Supplementary Material online.

**Fig. 4.**
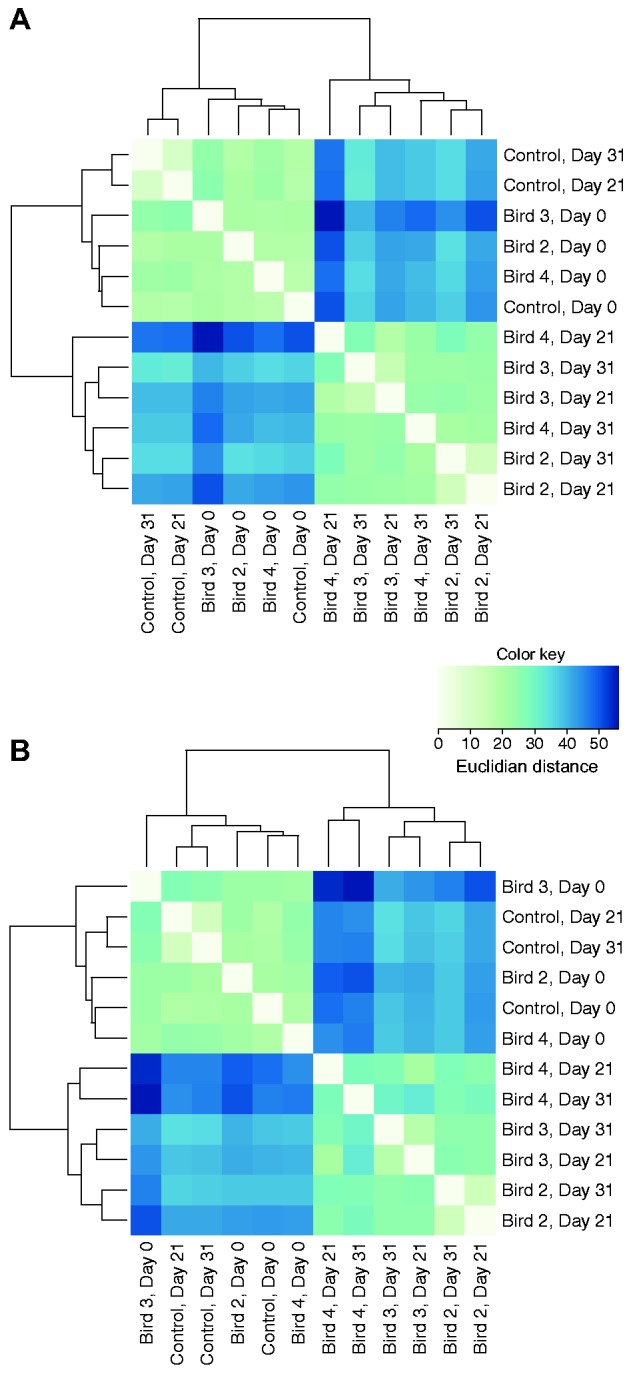
Similarities and differences across samples. Heat maps showing Euclidian distance of normalized gene expression between all 12 avian transcriptome samples for the significantly differentially expressed genes during (*A*) peak parasitemia and (*B*) decreasing parasitemia. A darker color indicates greater distances between samples. The dendrogram denotes hierarchical clustering of samples.

We also performed hierarchical clustering of samples based on expression similarities of the differentially expressed genes during peak and decreasing parasitemia (fig. 4) to better understand the quantitative differences between the two time points. All birds had highly similar expression profiles before infection (day 0), as well as the control bird during the whole experiment. The expression profiles of the peak and decreasing parasitemia stages did not form separate clades (fig. 4), further verifying the overall similarities found during the differential expression test. Instead samples clustered together by individual, with the exception of bird 4 during day 21. This individual experienced the highest parasitemia levels during day 21 (71.3%) (fig. 1*A*), and its expression profile during this time point is the most dissimilar sample compared with all uninfected samples (fig. 4*A*).

### Functions of Genes Responding to Malaria Infection

To gain a greater understanding of the biological and molecular processes that are involved in the response to malaria infection, we investigated the functions of the significantly differentially expressed genes. We used GSEA to test whether any GO terms were overrepresented among the significant genes during peak and decreasing parasitemia stages. The results of these analyses revealed that the differentially expressed genes were involved in the immune response, regulation of cell death, response to stress, telomerase activity, and metabolic and catabolic processes (see supplementary tables S4 and S5, Supplementary Material online, for full details of GO terms).

#### Immune Response

**Fig. 5.**
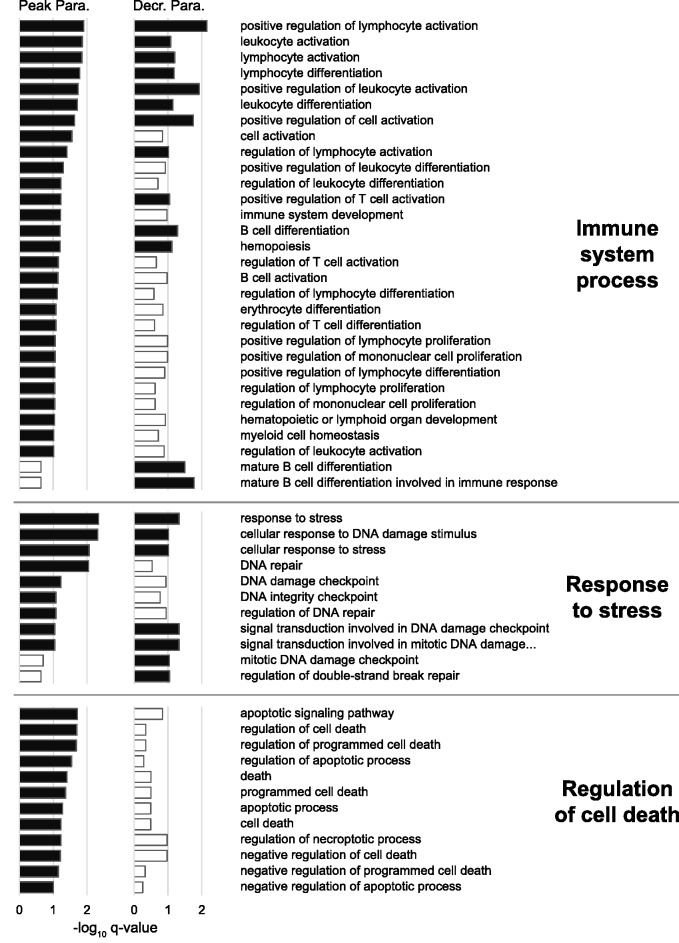
GO terms related to immune system, stress response, and cell death regulation overrepresented among significantly differentially expressed genes during peak and decreasing parasitemia. Black bars indicate significantly overrepresented functions (−log_10_ *q* value>1), and white bars represent nonsignificant *q* values. A higher −log_10_ *q* value denotes increased significance.

We found a large number of GO terms related to the parental term “immune system process” that were significantly overrepresented during peak parasitemia (*n* = 28) (fig. 5), for example, “positive regulation of T cell activation” and “lymphocyte differentiation.” This indicates that the birds’ immune system was highly active 21 days after infection during the peak parasitemia stage. By day 31, many of these immune functions had been switched off, or reduced in activity, as there were less than half the number of immune system functions overrepresented during decreasing parasitemia (*n* = 13; 46.4%). Activating immune responses is energetically and nutritionally costly (Lochmiller and Deerenberg 2000), and can induce immunopathological damage due to misdirected or overexpressed immune defenses (Sorci and Faivre 2009). Therefore, hosts seek to minimize the time immune processes are active. This is perhaps the reason why we see a reduced immune response in the birds during the later stage of infection. There were, however, two strongly significant overrepresented immune functions during the decreasing parasitemia stage that were not significant during peak parasitemia. Interestingly enough, they were both related to “mature B cell differentiation” (fig. 5). This is the process where a naive B cell acquires the specialized features of a mature or memory B cell during an immune response. Some immune genes were found significantly differentially expressed during both stages of infection. For example, *MR1* is a gene related to the major histocompatibility complex (MHC) class I, one of the most important and variable gene families of the adaptive immune system. This gene was significantly upregulated during peak parasitemia (*q* = 7.96e-3), and even more highly upregulated during decreasing parasitemia (*q* = 2.47e-5).

#### Response to Stress

Biological processes linked to the GO term “response to stress,” such as “DNA repair” and “cellular response to DNA damage stimulus,” were significantly overrepresented in infected birds (fig. 5). Different stress-related functions were activated during both parasitemia stages, although they were slightly more significant during peak parasitemia (fig. 5). During an infection, innate immune cells release reactive oxygen and nitrogen species to attack parasites (Sorci and Faivre 2009). These highly reactive nonspecific molecules target all living cells, capable of causing much damage to surrounding host cells and tissue. In addition, malaria parasites also generate cytotoxic compounds when digesting hemoglobin, which may cause further damage. Events like these may trigger oxidative stress responses. Previous research has found evidence of oxidative stress in hosts during malaria infection by measuring oxidant and antioxidant levels in the blood (see, e.g., Stocker et al. 1985; Erel et al. 1997; Becker et al. 2004; Sharma et al. 2012; Isaksson et al. 2013). Our study supports these findings and provides evidence of molecular regulation of oxidative stress in birds infected with malaria. For example, during peak parasitemia “glutathione transferase activity” was significantly overrepresented (*q* = 7.57e-4) (supplementary table S4, Supplementary Material online). The significant genes contributing to this term were all upregulated glutathione S-transferases (GSTs), which play critical roles in protecting cells from oxidative stress (Armstrong 1997; Hayes and McLellan 1999; Hayes and Strange 2000; Siritantikorn et al. 2007).

#### Cell Death Regulation

During peak parasitemia we found several significantly overrepresented functions related to “negative regulation of cell death” (fig. 5). Hosts often kill their own cells by programmed cell death (apoptosis) as a mean to control infections (Ashida et al. 2011). Here however, various mechanisms, such as apoptosis inhibitors, prevent cells from committing suicide. In contrast, not a single function related to cell death was found significantly overrepresented during the decreasing parasitemia stage (fig. 5). One explanation for why these functions are overrepresented during peak, but not decreasing levels of parasitemia, is that the birds may protect themselves against anemia. Because malaria parasites infect red blood cells, it is known that an infection can cause reduced levels of hematocrit and lead to anemia in the host (see, e.g., Swann and Kreier 1973; Chang and Stevenson 2004; Palinauskas et al. 2008; Cellier-Holzem et al. 2010). Alternatively, it is possible that *Plasmodium* is able to manipulate the host cell apoptosis regulatory mechanisms to its own benefit, in an attempt to evade the immune system. This strategy has been discovered in a wide variety of pathogens (reviewed in Lamkanfi and Dixit 2010; Ashida et al. 2011), including *Plasmodium* (van de Sand et al. 2005).

#### Telomerase Activity

Our analyses showed that the function “telomere maintenance via telomerase” was significantly overrepresented during peak parasitemia (*q* = 0.059) (supplementary table S4, Supplementary Material online). Telomeres are the essential structures located at the ends of eukaryotic chromosomes, protecting them from degradation, with telomerase being the principal enzyme involved in telomere lengthening. High rates of telomere shortening are an indicator of stress, and it has previously been shown that malaria parasites can increase the rate of telomere loss in infected birds (Asghar 2012). One of the significant genes in our study contributing to the overrepresentation of “telomere maintenance via telomerase” was *PINX1*, a potent telomerase inhibitor linked to telomere shortening (Zhou and Lu 2001). The upregulation of *PINX1* and other telomere-related genes indicated that telomerase activity was potentially reduced in birds with malaria during infection. In fact, as part of another study, the telomere lengths of the birds used here have been measured and it was found that the inoculated birds did indeed have reduced telomeres during infection (Asghar M et al., unpublished data).

#### Metabolic and Catabolic Processes

**Fig. 6.**
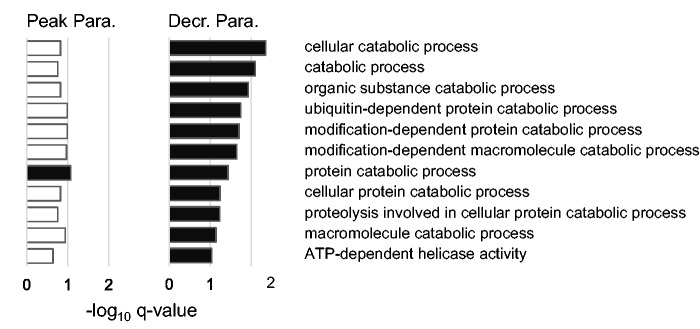
Catabolic processes are engaged only during the decreasing parasitemia stage. GO terms related to catabolic processes overrepresented among significantly differentially expressed genes during peak and decreasing parasitemia. Black bars indicate significantly overrepresented functions (−log_10_ *q* value>1), and white bars represent nonsignificant *q* values. A higher −log_10_ *q* value denotes increased significance.

Many functions related to metabolism and oxidation–reduction processes were found to be significantly overrepresented among the significant genes during both parasitemia stages (supplementary tables S4 and S5, Supplementary Material online). An infection induces a major shift in the metabolic priorities of the host (Lochmiller and Deerenberg 2000), and so the overrepresentation of metabolism-related activities in infected birds is not surprising. Metabolic processes have also been found to be of importance in other host species infected with malaria (see, e.g., Li et al. 2008; Albuquerque et al. 2009; Sengupta et al. 2011). Interestingly, however, is that catabolic processes were initiated in the later stages of malaria infection in our study, as only one GO term related to catabolic processes was overrepresented during peak parasitemia in comparison to 11 during the decreasing parasitemia stage (fig. 6).

#### Functions of Genes that Were Differentially Expressed between the Two Parasitemia Stages

The 23 genes significantly differentially expressed between day 21 and day 31 comprised seven microRNAs (miRNAs) and only four annotated genes (17.4%) (supplementary table S3, Supplementary Material online). The four annotated genes were *MKL2* and *CASQ2*, both genes involved in the heart muscle, *CLK2*, a gene involved in the regulation of RNA splicing, and finally *ARPC3*, which is included in the Fc-gamma receptor signaling pathway involved in phagocytosis. To better understand the biological differences between the two infected time points, we also evaluated the functions of the differentially expressed genes unique to each parasitemia stage (fig. 3) using GSEA. Significant genes that were unique to the peak parasitemia stage (*n* = 138) were found to be overrepresented in only one GO term, “*cis–trans* isomerase activity” (*q* = 0.037). Conversely, significant genes unique to the decreasing parasitemia stage (*n* = 449) were significantly overrepresented within the more general function “catabolic process” (*q* = 0.092), indicating once more that the increased activity of catabolic processes seems to be important during the later stages of malaria infection.

### RNA-seq Identifies Genes Involved in Host Response Regardless of Annotation Status

One advantage of using RNA-seq to measure gene expression, as opposed to, for example, microarray, is whole transcriptome identification, hence facilitating the discovery of novel and unannotated genes. Therefore, we evaluated the percentage of genes differentially expressed in response to malaria infection containing annotation of one or more GO terms. Of the 795 significant genes differentially expressed during peak parasitemia, 79.1% (*n* = 629) had GO annotations. Similarly, 82.7% (*n* = 915) of the 1,106 significant genes found in the decreasing parasitemia stage were annotated. Altogether, about 20% of the genes responding to malaria infection had unknown functions. This suggests that we would have greatly underestimated the avian host transcriptional response to malaria if we had used other molecular methods based only on known candidate genes. In fact, four of the top ten significant genes from the peak parasitemia stage were completely uncharacterized (table 1). These highly significant uncharacterized genes represent potential targets for future, more detailed research that may reveal novel host–parasite interactions.

**Fig. 7.**
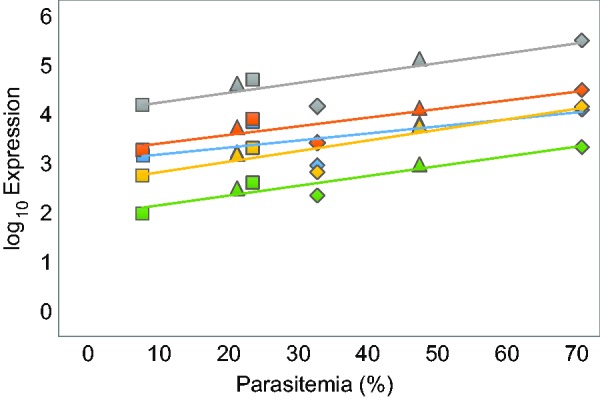
miRNA genes are highly significant and correlate with parasitemia levels. Normalized log-transformed expression levels of the top five significant miRNA genes (see table 1) against parasitemia levels in birds (% infected red blood cells) during the two infected time points (days 21 and 31). Colors represent different miRNA genes, and lines are fitted linear regression lines for each gene, corresponding to the same color. Spearman’s rank correlation tests: ENSTGUG00000018584 (green; *R*_s_ = 0.83; *n* = 6; *P* = 0.06), ENSTGUG00000018598 (yellow; *R*_s_ = 0.83; *n* = 6; *P* = 0.06), ENSTGUG00000017658 (blue; *R*_s_ = 0.61; *n* = 6; *P* = 0.20), ENSTGUG00000018618 (orange; *R*_s_ = 0.83; *n* = 6; *P* = 0.06), and ENSTGUG00000018567 (gray; *R*_s_ = 0.66; *n* = 6; *P* = 0.18). Symbols represent the different bird individuals; circle = bird 1 (control bird), square = bird 2 (low parasitemia), triangle = bird 3 (intermediate parasitemia), and diamond = bird 4 (high parasitemia).

More specifically, we observed that several unannotated miRNA genes were highly important in the host response, comprising five of the top ten most significant genes during both parasitemia stages (table 1). miRNAs are small regulatory RNA molecules principally involved in posttranscriptional silencing of mRNA. An increasing number of studies are now finding evidence that miRNAs have critical functions in the immune system (Lindsay 2008; Carissimi et al. 2009; Xiao and Rajewsky 2009). The top significant miRNA genes in our study had extremely low *q* values (8.6e-79) (table 1), high log_2_ fold changes (up to 8.0) (supplementary table S1, Supplementary Material online), and their expression levels were highly correlated with parasitemia levels (fig. 7). LaMonte et al. (2012) discovered that some miRNAs were involved in a unique host defense strategy against malaria parasites in humans. Sickle cell erythrocytes upregulated specific miRNAs which were subsequently translocated into *P. falciparum* where they integrated with essential parasite mRNA, inhibiting them from being translated (LaMonte et al. 2012). In addition, other reports are now testifying that several pathogens are able to control the host’s expression of miRNAs (Hakimi and Cannella 2011; Eulalio et al. 2012), including apicomplexan parasites such as *Plasmodium*. As there is no information available yet for the miRNAs and uncharacterized genes in our study, we cannot describe their role nor function; however, this will be possible to evaluate in subsequent studies as gene annotation continues to improve.

### Highly Conserved Gene Responses to Malaria across Vertebrates

It is currently unknown whether there are evolutionary conserved responses to *Plasmodium* infection across vertebrates due to a lack of information across diverse host species. We therefore compared the avian host response in our study with data on other studied host malaria systems—human and rodent malaria. Idaghdour et al. (2012) evaluated blood transcriptome responses of both children to *P. falciparum* and mice to *P. chabaudi*, using microarray techniques. From the supplementary data of Idaghdour et al. (2012), we downloaded genes significantly differentially expressed in response to malaria, of human (2,948 genes) and mouse (1,301 genes). These genes were then compared with our significantly differentially expressed bird genes (both parasitemia stages combined; *n* = 837 with gene names) to test whether any genes responding to malaria were the same in all three taxa. We identified 140 genes (16.7%) in common between birds and humans (supplementary table S8, Supplementary Material online), and 56 genes (6.7%) in common between birds and mice (supplementary table S9, Supplementary Material online). We also compared mouse genes to human genes, and found 240 genes (18.4%) shared between the two mammalian hosts. Thirteen genes were present in the transcriptional response to malaria in all three organisms (table 2). Of these 13 significant genes, several are highly involved in the immune defense and/or cell death regulation, for example, lymphocyte antigen 86, B cell CLL/lymphoma 6, and suppressor of cytokine signaling 3 (table 2). These results suggest that there are potentially highly conserved evolutionary immune responses activated by malaria infections which might provide valuable targets for research into disease control programs. We want to emphasize, however, that the overlap of gene response is based on only three species investigated by two studies, and should therefore be treated with caution. With future sequencing of more transcriptome responses to malaria, this will be possible to evaluate in greater detail.

**Table 2.** Genes Significantly Differentially Expressed during Malaria Infection that Are Shared across Birds, Humans, and Mice.

Gene Name	Gene Description	Bird FC	Human FC	Mouse FC
*ACOT7*	Acyl-CoA thioesterase 7	+	+	+
*SOCS3*	Suppressor of cytokine signaling 3	+	+	+
*VIM*	Vimentin	+	+	+
*ITM2B*	Integral membrane protein 2B	+	+	−
*PSME3*	Proteasome activator complex subunit 3	+	+	−
*SH3GLB1*	SH3-domain GRB2-like endophilin B1	+	+	−
*MRPL33*	Mitochondrial ribosomal protein L33	+	−	+
*AFG3L2*	AFG3 ATPase Family Gene 3-Like 2	−	−	−
*LY86*	Lymphocyte antigen 86	−	−	−
*MBNL2*	Muscleblind-like splicing regulator 2	−	−	−
*RPL23*	Ribosomal protein L23	−	−	+
*BCL6*	B cell CLL/lymphoma 6	−	+	−
*ANXA2*	Annexin A2	−	+	+

Note.—FC, log_2_ fold change direction of gene expression during infection (+, upregulated; −, downregulated).

## Conclusions

By using RNA-seq, we were able to evaluate the complete avian transcriptome response to malaria, and find expression changes occurring not only in well-documented, annotated genes but also in unannotated, uncharacterized genes. As a result this approach improves our understanding of the molecular and physiological processes occurring during different stages of malaria infections while at the same time allowing the potential discovery of novel interactions between hosts and parasites. Finally, by integrating information across divergent hosts we foresee that it will be possible to gain a much deeper understanding of the coevolution of molecular mechanisms underpinning host–parasite interactions.

## Materials and Methods

### Experimental Setup

The infection experiment was carried out in 2012 at the Biological Station of the Zoological Institute of the Russian Academy of Sciences on the Curonian Spit in the Baltic Sea (55 °05′N, 20 °44′E). Wild juvenile siskins were caught with mist nets in the summer and housed in aviaries. Experimental procedures in this study were approved by the International Research Co-operation Agreement between the Biological Station Rybachy of the Zoological Institute of the Russian Academy of Sciences and Institute of Ecology of Nature Research Centre (25th May 2010). All efforts were made to minimize handling time and potential suffering of birds. The full experimental procedure incorporated several birds and the results from the complete infection study is currently under preparation. Siskins have proven to be excellent study organisms for avian malaria experiments (Palinauskas et al. 2008, 2011). They are susceptible to several *Plasmodium* lineages, yet juvenile siskins caught early in the summer are uninfected. They are abundant at the study site and suitable to house in captivity as opposed to several other small wild birds. We used three inoculated birds and one control bird as part of this transcriptome study. Although the sample size is small (three replicates per group, with four uninfected birds at the start of the experiment and one additional bird for control), it provided us with enough variation to perform all analyses. This setup allowed us to follow the same individuals over the course of the experiment using a longitudinal design, minimizing interindividual variation in expression levels.

The inoculated birds were infected with a single injection of blood containing the erythrocytic stages of *P. ashfordi* (lineage GRW2), described by Valkiūnas et al. (2007). The strain we used was originally obtained from one naturally infected common cuckoo (*Cuculus canorus*) in 2011. It was subsequently multiplied in common crossbills (*Loxia curvirostra*) and then deep frozen in liquid nitrogen for storage. Prior to this experiment, a single crossbill was infected and served as donor of the infection. Crossbills were used because they are large enough to provide the amount of blood needed for injections. Importantly, they are also susceptible for this particular strain, free from other blood parasites, and suitable for keeping under laboratory conditions. By utilizing only one donor, we effectively made sure to distribute the same clonal strain and parasite quantity to the recipient birds.

All birds were originally screened for parasites with both molecular polymerase chain reaction (PCR) screening methods (details in Hellgren et al. 2004) and microscopic blood smear examination (described in Valkiūnas et al. 2008) to ensure no prior infection. A subinoculation of a freshly prepared mixture containing infected blood was made into the pectoral muscle of the recipient birds (details in Palinauskas et al. 2008). The control bird was injected at the same time as inoculated birds, but with blood from a donor crossbill free of parasites. The birds were observed continuously throughout the duration of the experiment. Blood for quantitative estimations of parasitemia levels was sampled before and throughout the experiment in heparinized microcapillaries by puncturing the brachial vein, and subsequently smeared on blood films for microscopic examinations and stored in SET-buffer for quantitative PCR. Blood for RNA-sequencing was collected at three time points in empty tubes and immediately frozen in liquid nitrogen and stored at −80 °C until extraction. For detailed procedures regarding the infection experiment, see methods in Palinauskas et al. (2008).

Samples from three time points were used for RNA-sequencing: Day 0 (before infection), day 21 postinfection (during peak parasitemia), and day 31 postinfection (decreasing parasitemia). The individual parasitemia levels of the infected birds varied substantially, with 24.0%, 48.0%, and 71.3% of red blood cells infected during peak parasitemia, and later 8.2%, 21.8%, and 33.3%, respectively, during the decreasing parasitemia stage (fig. 1*A*). All three birds would normally be classified as having very high parasitemia levels, though for the sake of clarity we have in this study referred to their relative levels: Bird 1 (control bird), bird 2 (low parasitemia), bird 3 (intermediate parasitemia), and bird 4 (high parasitemia).

### RNA Extraction and Sequencing

Total RNA was extracted from 20 µl of whole blood samples using 1,000 µl TRIzol LS Reagent (Invitrogen Carlsbad, CA) and homogenized using a vortex. The samples were then incubated in room temperature for 5 min before 200 µl of chloroform (Merck KGaA, Darmstadt, Germany) was added. Following another room temperature incubation for 3 min, the samples were centrifuged at 11,000 rpm for 17 min at 4 °C. The supernatant was then transferred to new tubes, and using an RNeasy Mini Kit (Qiagen, GmbH, Hilden, Germany), we followed the manufacturer’s protocol starting at point 2 by adding 1 volume of 70% ethanol to the lysate. Total extracted RNA was shipped on dry ice, to Beijing Genomics Institute (BGI), China, for RNA quality control, DNAse treatment, rRNA reduction, and amplification using the SMARTer Ultra Low kit (Clontech Laboratories, Inc.). BGI performed library preparation, cDNA synthesis, and paired-end sequencing using Illumina HiSeq 2000. Raw reads were initially filtered with the Illumina chastity filter, although we quality-screened the reads an additional time using FastQC (v. 0.10.1) (http://www.bioinformatics.babraham.ac.uk/projects/fastqc/). A combined total of 720 million paired-end reads, partitioned into 90-bp-long reads (392 million), and 65-bp-long reads (328 million) passed read quality control and filtering (supplementary table S10, Supplementary Material online).

### Read Mapping

Quality filtered reads were mapped with TopHat2 (v. 2.0.9) (Kim et al. 2013), which makes use of the aligner Bowtie2 (v. 2.1.0) (Langmead and Salzberg 2012), to the unmasked zebra finch (*Taeniopygia guttata*) genome (v. 3.2.4.73) (Warren et al. 2010), obtained from Ensembl (v. 73) (Flicek et al. 2013). Reads were allowed a maximum mismatch rate of 20% and were guided in TopHat2 using the zebra finch transcriptome (v. 3.2.4.73) (Flicek et al. 2013). By using a genome-guided approach instead of a de novo assembly of reads, we effectively made sure to discriminate from any reads originating from the malaria parasite in the analyses.

Five hundred and forty million unique reads were successfully mapped to the genome (supplementary table S10, Supplementary Material online). Two main factors will reduce the proportion of sequence reads mapped in this study. First, a substantial part of the reads in infected birds originates from the parasite’s mRNA and hence will lower the proportion of avian reads. This can be seen in the reduced percentage of reads mapped in infected birds compared with the same bird before infection, day 0 (supplementary table S10, Supplementary Material online). Second, the bird species sequenced was Eurasian siskin, and even though avian genomes are highly conserved (Ellegren 2010), use of the zebra finch genome will automatically reduce mapping statistics due to evolutionary divergence between the two species. Taking these two factors into account, the percentage of reads mapped to the genome was nonetheless relatively high (84.1% for 90-bp reads and 64.2% for 65-bp reads). Average base pair coverage per individual relative to the zebra finch entire transcriptome was high (137.5) (supplementary table S10, Supplementary Material online), despite sequencing only blood and therefore we expected low or no coverage from tissue-specific genes. Reads used in this study have been uploaded to the NCBI Sequence Read Archive under the accession number SRP045347.

### Principal Component Analysis

Unique reads that mapped unambiguously to genes were counted using HTSeq (v. 0.5.3p9) (Anders et al. 2014) and SAMtools (v. 0.1.19) (Li et al. 2009), and subsequently analyzed in DESeq2 (Love et al. 2014). In order to avoid any bias of experimental design, variance stabilized transformation of counts (explained in Anders and Huber 2010) was used in DESeq2 to evaluate sample clustering and sample distances (fig. 4), and the PCA (fig. 1*B*). The PCA was performed on all genes and all individuals concurrently, with 28.4% of the variance explained by PC1 and 20.1% by PC2 (fig. 1*B*). The PC1 represents expression differences seen in the birds due to the experimental procedure. By injecting all birds with blood they potentially experienced a minor immune response directly after injection at day 0. However, mean survival time of red blood cells after heterologous blood transfusion in birds has been estimated to only 0.1–2.6 days (Sandmeier et al. 1994; Degernes et al. 1999). It is therefore highly unlikely the response to blood injection would still be present during sampling 21 and 31 days later. Instead, PC1 most likely represent the birds’ response to being caught, handled, and having blood collected for both RNA-seq and parasitemia estimations. The act of catching wild birds and housing them in captivity induces a wide range of different environmental stresses and stimuli (Morgan and Tromborg 2007; Dickens et al. 2009). Taken together, these factors are likely to affect expression levels in a large number of genes, whereas infection triggers changes in fewer, more targeted genes. This is supported by the observation that all four birds (regardless of parasitemia levels) moved in the same direction and with similar magnitude on PC1 from day 0 to days 21 and 31 of the experiment (fig. 1*C* and *D*). Additionally, different bird individuals were found interspersed on PC1 (fig. 1*B*), indicating broad underlying baseline differences in the blood transcriptomes between individuals, which is expected. Our interpretation of PC1 as the experimental procedure response is strongly reinforced by evidence from GSEA, where functions related to stress, coagulation, and wound healing can be seen in significant genes contributing more to the loading of samples on PC1 than PC2 (supplementary tables S6 and S7, Supplementary Material online).

### Differential Gene Expression Analyses

Differential gene expression analyses were performed with DESeq2 (v. 1.0.19) (Anders and Huber 2010; Love et al. 2014), in R (v. 3.0.2) (R Core Team 2014), using a pairwise design with individual as a blocking factor. Plots were made using ggplot2 (Wickham 2009). DESeq2 is suitable for studies with fewer replicates, and consistently shows high sensitivity and precision (Love et al. 2014). In this software, counts are normalized for library size differences using the geometric mean, and modeled with a negative binomial distribution. While earlier methods based on a Poisson distribution model underestimate the effect of biological variability, the negative binomial distribution model tests for overdispersion and makes fewer type I errors (Anders and Huber 2010). Shrinkage of dispersion and fold changes estimates are implemented in DESeq2 to improve stability and interpretability (Love et al. 2014). In practice, this means that logarithmic fold changes will have stronger shrinkage when there is little information available for a gene (i.e., low read count, high dispersion, or few degrees of freedom). Differentially expressed genes were corrected for false positives using the Benjamini and Hochberg false discovery rate (FDR) correction for multiple testing (Benjamini and Hochberg 1995), and the corrected *P* values are reported throughout the article as *q* values. Underlying data distribution has been depicted using density curves and MA plots in supplementary figures S3 and S4, Supplementary Material online.

Several strict filtering criteria were used in DESeq to ensure that we only examined genes which changed expression in response to malaria infection. First, for every differential expression test, only the genes with a combined mean count of ≥5, *P* < 0.05, and FDR (*q* value) < 0.1 were classified as significant. This resulted in an initial set of 1,230 genes (during peak parasitemia) and 1,784 genes (during decreasing parasitemia) significantly differentially expressed. Second, in order to tease apart the effects of infection from the effects of the experiment, we removed from the set of significant genes, all genes that had a larger PC1 than PC2 score. The reasoning behind this screening was because the PC2 dimension explained expression variance present in the birds due to malaria infection, and PC1 explained variance due to the experimental procedure (see previous paragraph about PCA). By using strict selection criteria in our analyses and scrutinizing all significant genes with higher PC1 scores, we miss the genes that changed expression levels both in response to malaria infection and in response to experimentation, simultaneously, provided that their experiment response was higher than their infection response. It is therefore important to note that these results are likely to represent more specific responses to malaria infection and may underestimate the importance of general immune mechanisms and stress responses that are also activated by handling the birds and collecting their blood.

### Validation of Differential Gene Expression Results

Validation of gene expression results was examined by employing a second method testing for differential gene expression using edgeR (v. 3.4.2) (Robinson et al. 2010). In edgeR, infected samples at days 21 and 31 postinfection were tested separately against the uninfected samples at day 0, using a pairwise multifactorial design with bird individual as blocking factor. Similar to the analyses in DESeq2, we only accepted genes as significant if they passed the following criteria: Mean counts per million ≥ 0.1, *P* < 0.05, and FDR < 0.1. Subsequent filtering of significant genes with higher PC2 scores ensured we were only using genes responding to the malaria infection, with the same underlying reasoning as in the DESeq2 analyses. edgeR discovered slightly fewer differentially expressed genes responding to malaria than DESeq2; 603 genes during peak parasitemia and 939 during decreasing parasitemia. However, most of the significant genes (88.7%) were shared by both methods (supplementary fig. S5, Supplementary Material online), strongly validating our initial results.

### Gene Set Enrichment Analyses

Functional annotations and overrepresentation of GO terms (Ashburner et al. 2000) were analyzed and visualized in Cytoscape (v. 3.1.0) (Cline et al. 2007), using BiNGO (v. 3.0.2) (Maere et al. 2005). Full ontology file was downloaded from www.geneontology.org (v. 2014-04-15). The gene enrichment analyses were performed using a hypergeometric test for overrepresentation and corrected for false positives with the Benjamini and Hochberg FDR correction (Benjamini and Hochberg 1995). We evaluated GO terms from the domains biological processes and molecular functions. Functional groups with a FDR (*q*-value) < 0.1 were regarded as statistically significantly overrepresented.

## Supplementary Material

Supplementary tables S1–S10 and figures S1–S5 are available at *Molecular Biology and Evolution* online (http://www.mbe.oxfordjournals.org/).

Supplementary Data

## References

[msv016-B1] Albuquerque SS, Carret C, Grosso AR, Tarun AS, Peng X, Kappe SHI, Prudêncio M, Mota MM (2009). Host cell transcriptional profiling during malaria liver stage infection reveals a coordinated and sequential set of biological events. BMC Genomics.

[msv016-B2] Anders S, Huber W (2010). Differential expression analysis for sequence count data. Genome Biol..

[msv016-B3] Anders S, Pyl PT, Huber W (2014). HTSeq—a Python framework to work with high-throughput sequencing data. Bioinformatics.

[msv016-B4] Armstrong R (1997). Structure, catalytic mechanism, and evolution of the glutathione transferases. Chem Res Toxicol..

[msv016-B5] Asghar M (2012).

[msv016-B6] Ashburner M, The Gene Ontology Consortium (2000). Gene Ontology: tool for the unification of biology. Nat Genet..

[msv016-B7] Ashida H, Mimuro H, Ogawa M, Kobayashi T, Sanada T, Kim M, Sasakawa C (2011). Cell death and infection: a double-edged sword for host and pathogen survival. J Cell Biol..

[msv016-B8] Atkinson CT, Dusek RJ, Woods KL, Iko WM (2000). Pathogenicity of avian malaria in experimentally-infected Hawaii Amakihi. J Wildl Dis..

[msv016-B9] Atkinson CT, LaPointe DA (2009). Introduced avian diseases, climate change, and the future of Hawaiian honeycreepers. J Avian Med Surg..

[msv016-B10] Atkinson CT, Woods KL, Dusek RJ, Sileo LS, Iko WM (1995). Wildlife disease and conservation in Hawaii: pathogenicity of avian malaria (*Plasmodium relictum*) in experimentally infected Iiwi (*Vestiaria coccinea*). Parasitology.

[msv016-B11] Becker K, Tilley L, Vennerstrom JL, Roberts D, Rogerson S, Ginsburg H (2004). Oxidative stress in malaria parasite-infected erythrocytes: host-parasite interactions. Int J Parasitol..

[msv016-B12] Benjamini Y, Hochberg Y (1995). Controlling the false discovery rate: a practical and powerful approach to multiple testing. J R Stat Soc Ser B..

[msv016-B13] Bensch S, Hellgren O, Pérez-Tris J (2009). MalAvi: a public database of malaria parasites and related haemosporidians in avian hosts based on mitochondrial cytochrome b lineages. Mol Ecol Resour..

[msv016-B14] Bensch S, Pérez-Tris J, Waldenström J, Hellgren O (2004). Linkage between nuclear and mitochondrial DNA sequences in avian malaria parasites: multiple cases of cryptic speciation?. Evolution.

[msv016-B15] Bensch S, Waldenström J, Jonzén N, Westerdahl H, Hansson B, Sejberg D, Hasselquist D (2007). Temporal dynamics and diversity of avian malaria parasites in a single host species. J Anim Ecol..

[msv016-B16] Carissimi C, Fulci V, Macino G (2009). MicroRNAs: novel regulators of immunity. Autoimmun Rev..

[msv016-B17] Cellier-Holzem E, Esparza-Salas R, Garnier S, Sorci G (2010). Effect of repeated exposure to *Plasmodium relictum* (lineage SGS1) on infection dynamics in domestic canaries. Int J Parasitol..

[msv016-B18] Chang K-H, Stevenson MM (2004). Malarial anaemia: mechanisms and implications of insufficient erythropoiesis during blood-stage malaria. Int J Parasitol..

[msv016-B19] Cline MS, Smoot M, Cerami E, Kuchinsky A, Landys N, Workman C, Christmas R, Avila-Campilo I, Creech M, Gross B (2007). Integration of biological networks and gene expression data using Cytoscape. Nat Protoc..

[msv016-B20] Cox FE (2010). History of the discovery of the malaria parasites and their vectors. Parasit Vectors..

[msv016-B21] Degernes LA, Croiser ML, Harrison LD, Dennis PM, Diaz DE (1999). Autologous, homologous and heterologous red blood cell transfusions in cockatiels (*Nymphicus hollandicus*). J Avian Med Surg..

[msv016-B22] Dickens MJ, Earle KA, Romero LM (2009). Initial transference of wild birds to captivity alters stress physiology. Gen Comp Endocrinol..

[msv016-B23] Ellegren H (2010). Evolutionary stasis: the stable chromosomes of birds. Trends Ecol Evol..

[msv016-B24] Erel O, Kocyigit A, Avci S, Aktepe N, Bulut V (1997). Oxidative stress and antioxidative status of plasma and erythrocytes in patients with vivax malaria. Clin Biochem..

[msv016-B25] Eulalio A, Schulte L, Vogel J (2012). The mammalian microRNA response to bacterial infections. RNA Biol..

[msv016-B26] Flicek P, Ahmed I, Amode MR, Barrell D, Beal K, Brent S, Carvalho-Silva D, Clapham P, Coates G, Fairley S (2013). Ensembl 2013. Nucleic Acids Res..

[msv016-B27] Garnham PCC (1966). Malaria parasites and other Haemosporidia.

[msv016-B28] Hakimi M, Cannella D (2011). Apicomplexan parasites and subversion of the host cell microRNA pathway. Trends Parasitol..

[msv016-B29] Hayes JD, McLellan LI (1999). Glutathione and glutathione-dependent enzymes represent a co-ordinately regulated defence against oxidative stress. Free Radic Res..

[msv016-B30] Hayes JD, Strange RC (2000). Glutathione S-transferase polymorphisms and their biological consequences. Pharmacology.

[msv016-B31] Hellgren O, Pérez-Tris J, Bensch S (2009). A jack-of-all-trades and still a master of some: prevalence and host range in avian malaria and related blood parasites. Ecology.

[msv016-B32] Hellgren O, Waldenström J, Bensch S (2004). A new PCR assay for simultaneous studies of *Leucocytozoon*, *Plasmodium*, and *Haemoproteus* from avian blood. J Parasitol..

[msv016-B33] Idaghdour Y, Quinlan J, Goulet J-P, Berghout J, Gbeha E, Bruat V, de Malliard T, Grenier J-C, Gomez S, Gros P (2012). Evidence for additive and interaction effects of host genotype and infection in malaria. Proc Natl Acad Sci U S A..

[msv016-B34] Isaksson C, Sepil I, Baramidze V, Sheldon BC (2013). Explaining variance of avian malaria infection in the wild: the importance of host density, habitat, individual life-history and oxidative stress. BMC Ecol..

[msv016-B35] Kim D, Pertea G, Trapnell C, Pimentel H, Kelley R, Salzberg SL (2013). TopHat2: accurate alignment of transcriptomes in the presence of insertions, deletions and gene fusions. Genome Biol..

[msv016-B36] Lamkanfi M, Dixit VM (2010). Manipulation of host cell death pathways during microbial infections. Cell Host Microbe..

[msv016-B37] LaMonte G, Philip N, Reardon J, Lacsina JR, Majoros W, Chapman L, Thornburg CD, Telen MJ, Ohler U, Nicchitta CV (2012). Translocation of sickle cell erythrocyte microRNAs into *Plasmodium falciparum* inhibits parasite translation and contributes to malaria resistance. Cell Host Microbe..

[msv016-B38] Langmead B, Salzberg SL (2012). Fast gapped-read alignment with Bowtie 2. Nat Methods..

[msv016-B39] Levine ND (1988). The protozoan phylum Apicomplexa.

[msv016-B40] Li H, Handsaker B, Wysoker A, Fennell T, Ruan J, Homer N, Marth G, Abecasis G, Durbin R (2009). The Sequence Alignment/Map format and SAMtools. Bioinformatics.

[msv016-B41] Li J, Wang Y, Saric J (2008). Global metabolic responses of NMRI mice to an experimental *Plasmodium berghei* infection. J Proteome Res..

[msv016-B42] Lindsay MA (2008). microRNAs and the immune response. Trends Immunol..

[msv016-B43] Lochmiller RL, Deerenberg C (2000). Trade-offs in evolutionary immunology: just what is the cost of immunity?. Oikos.

[msv016-B44] Love MI, Huber W, Anders S (2014). Moderated estimation of fold change and dispersion for RNA-seq data with DESeq2. Genome Biol..

[msv016-B45] Maere S, Heymans K, Kuiper M (2005). BiNGO: a Cytoscape plugin to assess overrepresentation of gene ontology categories in biological networks. Bioinformatics.

[msv016-B46] Morgan KN, Tromborg CT (2007). Sources of stress in captivity. Appl Anim Behav Sci..

[msv016-B47] Oshlack A, Robinson MD, Young MD (2010). From RNA-seq reads to differential expression results. Genome Biol..

[msv016-B48] Palinauskas V, Valkiūnas G, Bolshakov CV, Bensch S (2008). *Plasmodium relictum* (lineage P-SGS1): effects on experimentally infected passerine birds. Exp Parasitol..

[msv016-B49] Palinauskas V, Valkiūnas G, Bolshakov CV, Bensch S (2011). *Plasmodium relictum* (lineage SGS1) and *Plasmodium ashfordi* (lineage GRW2): the effects of the co-infection on experimentally infected passerine birds. Exp Parasitol..

[msv016-B50] R Core Team (2014). http://www.R-project.org/.

[msv016-B51] Råberg L, Graham AL, Read AF (2009). Decomposing health: tolerance and resistance to parasites in animals. Philos Trans R Soc Lond B Biol Sci..

[msv016-B52] Ricklefs RE, Fallon SM (2002). Diversification and host switching in avian malaria parasites. Proc Biol Sci..

[msv016-B53] Robinson MD, McCarthy DJ, Smyth GK (2010). edgeR: a Bioconductor package for differential expression analysis of digital gene expression data. Bioinformatics.

[msv016-B54] Sandmeier P, Stauber EH, Wardrop KJ, Washizuka A (1994). Survival of pigeon red blood cells after transfusion into selected raptors. J Am Vet Med Assoc..

[msv016-B55] Sengupta A, Ghosh S, Basant A, Malusare S, Johri P, Pathak S, Sharma S, Sonawat HM (2011). Global host metabolic response to *Plasmodium vivax* infection: a 1H NMR based urinary metabonomic study. Malar J..

[msv016-B56] Sharma L, Kaur J, Shukla G (2012). Role of oxidative stress and apoptosis in the placental pathology of *Plasmodium berghei* infected mice. PLOS One.

[msv016-B57] Siritantikorn A, Johansson K, Ahlen K, Rinaldi R, Suthiphongchai T, Wilairat P, Morgenstern R (2007). Protection of cells from oxidative stress by microsomal glutathione transferase 1. Biochem Biophys Res Commun..

[msv016-B58] Sorci G, Faivre B (2009). Inflammation and oxidative stress in vertebrate host-parasite systems. Philos Trans R Soc Lond B Biol Sci..

[msv016-B59] Stocker R, Hunt NH, Buffinton GD, Weidemann MJ, Lewis-Hughes PH, Clark IA (1985). Oxidative stress and protective mechanisms in erythrocytes in relation to *Plasmodium vinckei* load. Proc Natl Acad Sci U S A..

[msv016-B60] Swann A, Kreier J (1973). *Plasmodium gallinaceum*: mechanisms of anemia in infected chickens. Exp Parasitol..

[msv016-B61] Valkiūnas G (2005). Avian malaria parasites and other haemosporidia.

[msv016-B62] Valkiūnas G, Iezhova TA, Krizanauskiene A, Palinauskas V, Sehgal RNM, Bensch S (2008). A comparative analysis of microscopy and PCR-based detection methods for blood parasites. J Parasitol..

[msv016-B63] Valkiūnas G, Zehtindjiev P, Hellgren O, Ilieva M, Iezhova TA, Bensch S (2007). Linkage between mitochondrial cytochrome b lineages and morphospecies of two avian malaria parasites, with a description of *Plasmodium* (*Novyella*) *ashfordi* sp. nov. Parasitol Res..

[msv016-B64] van Riper CI, van Riper SG, Goff ML, Laird M (1986). The epizootiology and ecological significance of malaria in Hawaiian land birds. Ecol Monogr..

[msv016-B65] van de Sand C, Horstmann S, Schmidt A, Sturm A, Bolte S, Krueger A, Lütgehetmann M, Pollok J-M, Libert C, Heussler VT (2005). The liver stage of *Plasmodium berghei* inhibits host cell apoptosis. Mol Microbiol..

[msv016-B66] Wang Z, Gerstein M, Snyder M (2009). RNA-Seq: a revolutionary tool for transcriptomics. Nat Rev Genet..

[msv016-B67] Warren WC, Clayton DF, Ellegren H, Arnold AP, Hillier LW, Künstner A, Searle S, White S, Vilella AJ, Fairley S (2010). The genome of a songbird. Nature.

[msv016-B68] Westermann AJ, Gorski SA, Vogel J (2012). Dual RNA-seq of pathogen and host. Nat Rev Microbiol..

[msv016-B69] White NJ, Turner GDH, Medana IM, Dondorp AM, Day NPJ (2010). The murine cerebral malaria phenomenon. Trends Parasitol..

[msv016-B70] WHO (World Health Organization) (2013). World malaria report 2013.

[msv016-B71] Wickham H (2009). ggplot2: elegant graphics for data analysis.

[msv016-B72] Xiao C, Rajewsky K (2009). MicroRNA control in the immune system: basic principles. Cell.

[msv016-B73] Zhou XZ, Lu KP (2001). The Pin2/TRF1-interacting protein PinX1 is a potent telomerase inhibitor. Cell.

