# Supplementary material for: The Avian Transcriptome Response to Malaria Infection
Source: Mol Biol Evol. 2015 Jan 29;32(5):1255–67. doi: 10.1093/molbev/msv016 (PMC4408411; doi:10.1093/molbev/msv016)
Supplement: Supplementary Data [file supp_32_5_1255__index.html]

The Avian Transcriptome Response to Malaria Infection — The Avian Transcriptome Response to Malaria Infection — Supplementary Data 

# The Avian Transcriptome Response to Malaria Infection

## Supplementary Data

files

**Files in this Data Supplement:**

- Supplementary Data - pdf file
- Supplementary Data - xlsx file
- Supplementary Data - xlsx file
- Supplementary Data - xlsx file
- Supplementary Data - xlsx file
