# Supplementary material for: The Avian Transcriptome Response to Malaria Infection
Source: Mol Biol Evol. 2015 Jan 29;32(5):1255–67. doi: 10.1093/molbev/msv016 (PMC4408411; doi:10.1093/molbev/msv016)
Supplement: Supplementary Data [file supp_msv016_Videvall_Supplementary_Figures_S1-5.pdf]

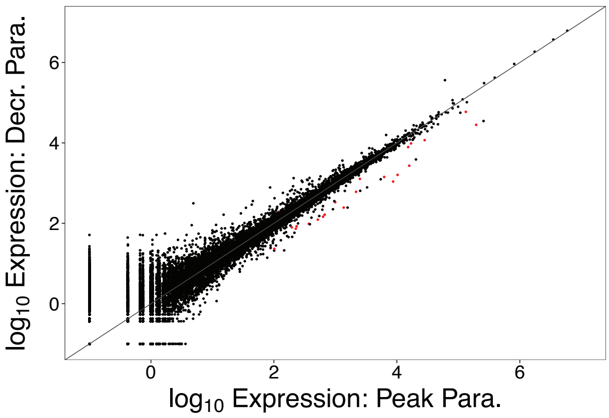

Supplementary Figure S1. Differential gene expression analysis between peak and decreasing parasitemia stages. Scatter plot of mean normalized gene expression levels (+ 0.1) in infected birds on a $\log_{10}$ scale comparing peak parasitemia against the decreasing parasitemia stage. All genes in the transcriptome are shown as points (n = 18 618), with non-significant genes in black and significantly differentially expressed genes in red.

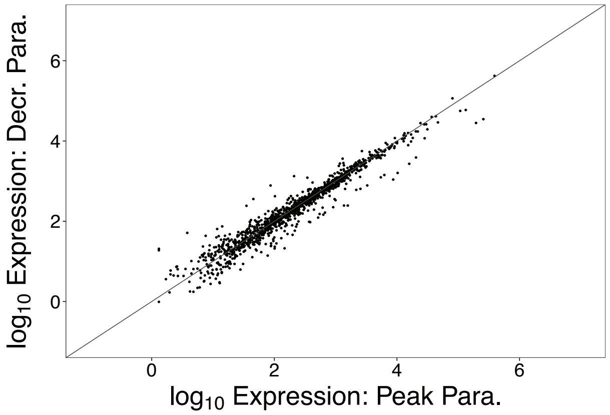

Supplementary Figure S2. Expression levels of the significant genes during peak and decreasing parasitemia. Scatter plot of mean normalized expression levels (+ 0.1) of all the significant genes during infection in inoculated birds (n = 1244) on a log_{10} scale comparing peak parasitemia against decreasing parasitemia.

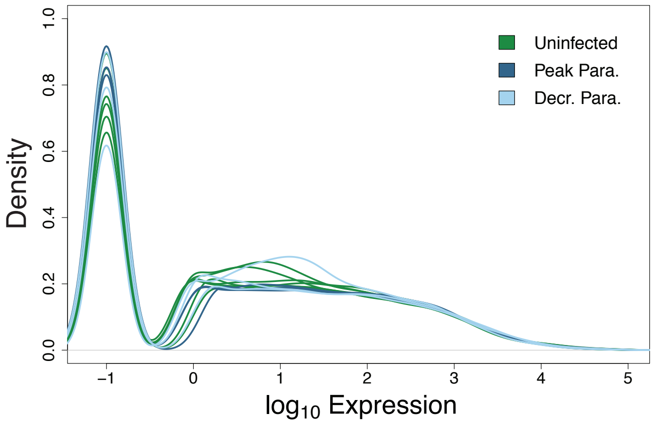

Supplementary Figure S3. Density curves for all samples. Density of mean normalized gene expression levels (+ 0.1) for each sample on a $\log_{10}$ scale. Uninfected samples are shown in green, infected samples during peak parasitemia in dark blue, and infected samples during decreasing parasitemia in light blue.

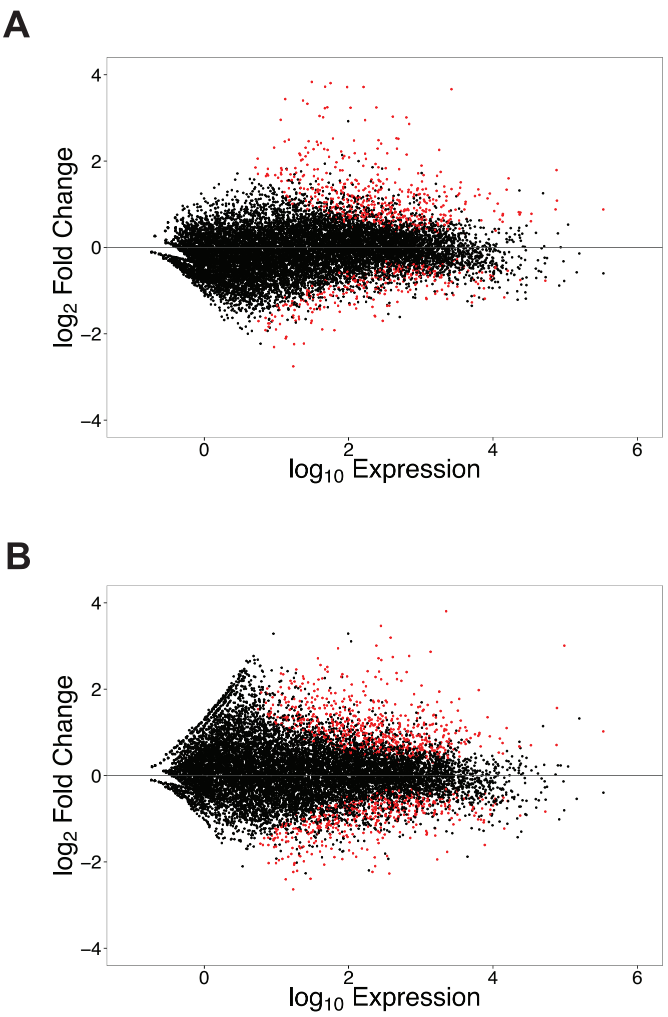

Supplementary Figure S4. MA plots of peak and decreasing parasitemia stages. MA plots comparing mean normalized gene expression levels (+ 0.1) to $\log_2$ fold changes during (**A**) peak parasitemia and (**B**) decreasing parasitemia. All genes in the transcriptome are shown as points (n = 18 618), with non-significant genes in black and significantly differentially expressed genes in red.

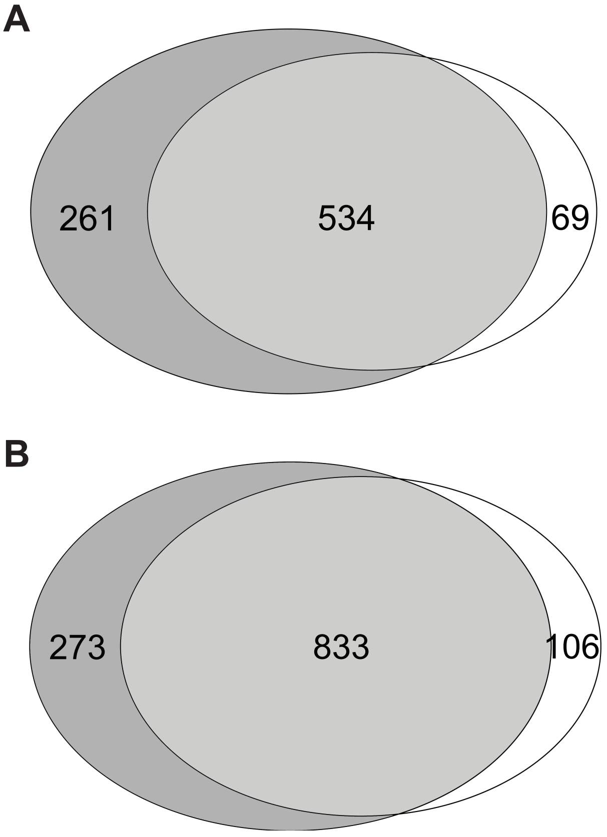

Supplementary Figure S5. Overlap of significantly differentially expressed genes derived from two different programs. Venn diagrams indicating overlap of significantly differentially expressed genes found in DESeq2 (dark grey) and edgeR (white), during (**A**) peak parasitemia and (**B**) decreasing parasitemia.
